# Dynamic Expression Pattern of SERPINA1 Gene from Duck (Anas platyrhynchos)

**DOI:** 10.1155/2019/1321287

**Published:** 2019-03-19

**Authors:** Tiantian Gu, Ningzhao Wu, Yang Zhang, Yu Huang, Jinping Du, Lizhi Lu, Xinsheng Wu, Qi Xu, Guohong Chen

**Affiliations:** ^1^Key Laboratory of Animal Genetics & Breeding and Molecular Design of Jiangsu Province, Yangzhou University, Yangzhou, 225009, China; ^2^Institute of Animal Science, Fujian Academy of Agricultural Sciences, Fujian, 350000, China; ^3^Institute of Animal Science, Hubei Academy of Agricultural Sciences, Wuhan, 430000, China; ^4^Institute of Animal Science, Zhejiang Academy of Agricultural Sciences, Hanghzou, 310000, China

## Abstract

SERPINA1 is a member of serine protease inhibitors and is increasingly considered to be a regulator of innate immunity in human and animals. However, the expression and function of SERPINA1 gene in immune defense against viral infection remain unknown in ducks. The full-length* du SERPINA1* cDNA sequence was obtained using reverse transcription polymerase chain reaction (RT-PCR) and rapid amplification of cDNA ends (RACE). It contained 1457 nucleotide, including 47-bp 5' UTR, 135-bp 3' UTR, and 1275-bp open reading frame (ORF), and encodes a 424-amino acid protein. Then, the tissue expression profile of du SERPINA1 gene was determined. Real-time quantitative polymerase chain reaction (real-time qPCR) analysis revealed that du* SERPINA1* mRNA is ubiquitous in various tissues, but higher expression levels were observed in lung and liver tissues. In addition, the expression pattern was investigated when the ducklings were challenged with duck hepatitis virus 1(DHV-1) and polyriboinosinic polyribocytidylic acid (poly I:C). After DHV-1 injection or poly I:C treatment, du* SERPINA1* mRNA was up-regulated in the liver and kidney tissues. However, the peak time in two tissues was not consistent. In kidney, the expression lever of* SERPINA1* increased immediately after the treatment while in liver tissue it kept steady until 12 h post-infection. Our results indicate that SERPINA1 has an active role in the antiviral response, and thus improve our understanding of the role of this protein.

## 1. Introduction

Serpins, as a superfamily of serine protease inhibitors, play a vital role in complement regulation inflammation, angiogenesis, tumor suppression, apoptosis and other physiological processes [1, 2]. There is ample clinical evidence that mutation in this gene could cause emphysema or liver disease, which showed a serious impact on the function and homeostasis of tissues and organs [3]. So far, sixteen clades have been identified, designated A through P, with an additional 10 serpins that are unclassified “orphans” [1].* SERPINA1*, also known as Alpha-1 anti-trypsin (AAT), is a vital member of the SERPIN superfamily, which plays an important role in anti-inflammatory properties [4–6]. Recent findings indicate that Alpha1-antitrypsin may not only prevent damage from proteolysis but may also specifically degrade elastin in tissues and organs, and inhibit some immune pathways to affect regulation of innate immunity [4–6].

Ducks, as a feasible model, play an important role in studying avian influenza and human hepatitis, and has raised interest in the duck immune system [7]. DHV-1 is a small RNA virus causing high mortality in ducks (Anas platyrhynchos), especially in younger ducklings. In order to reduce the effect of DHV-1, a large number of studies have been reported on this virus [8–14]. In our previous study, we identified differentially expressed sequence tags (ESTs) of* SERPINA1* using a suppression subtractive hybridization (SSH) cDNA library of 3-day-old ducklings treated with DHV-1 [15]. Our study showed that during DHV-1 and poly I:C infection, the expression of* SERPINA1* mRNA was up-regulated. In this study, we aim to expand those preliminary results, by assessing tissue-specific gene expression and the dynamic expression change of the SERPINA1 gene against the virus and thereby provide a theoretical basis for future immune pathological studies.

## 2. Materials and Methods

### 2.1. Ethics Approval and Consent to Participate

The animal experiment was reviewed and approved by the Institutional Animal Care and Use Committee of Yangzhou University (approval number: 151-2014). Procedures were performed in accordance with the Regulations for the Administration of Affairs Concerning Experimental Animals (Yangzhou University, China, 2012) and the Standards for the Administration of Experimental Practices (Jiangsu, China, 2008). We also confirm that all efforts were made to minimize suffering.

### 2.2. Ducks, Challenge Experiments, and Sample Collection

The 120 three-day-old domestic ducklings (Jingding duck) were purchased from the Chinese Waterfowl Germplasm Resource Pool (Taizhou, China). RT-PCR was used to make sure that the ducklings had not been exposed to DHV previous to our study [16]. Then, the ducklings were randomly divided into three groups, the 40 ducklings were injected with 0.4 mL of DHV-1(ELD50 10^−4.6^/0.2ml) according to our earlier trials [17, 18], and the 40 ducklings were injected with 0.4 mL of poly I: C (0.5 mg/mL, Invivogen, California, USA), another 40 treated with normal saline (as uninfected controls). Injection dose and injection method are consistent with our earlier trials [18]. Besides, the ducklings after the infection showed the typical symptoms of hepatitis by carrying the DHV-1 virus, and the relative results have been published [17]. In this study, at various times (0, 4, 8, 12, 24, 36, 48, 72, and 96 hours post-infection (h.p.i), three birds in each group were euthanized by injecting sodium pentobarbital (150 mg/kg) and killed by exsanguination. Total RNA was extracted from liver and kidney using the Trizol reagent (Invitrogen, California, USA). The results of clinical symptoms and autopsy were recorded.

Additionally, five healthy three-day-old domestic ducks (Jingding duck,* Anas platyrhynchos*) were purchased from the Chinese Waterfowl Germplasm Resource Pool (Taizhou, China). Tissues, including liver, spleen, lung, heart, kidney, thymus, breast muscle and leg muscle, were obtained after euthanasia that the ducklings were immediately anesthetized with sodium pentobarbital (intraperitoneal injection; 150 mg/kg) and killed by exsanguination. These birds were referred to as morbid ducklings. The tissues were snap-frozen in liquid nitrogen immediately and stored at -80°C.

### 2.3. RNA Extraction and Cloning of du SERPINA1 cDNA

Total RNA was isolated from the liver of ducks with TRIzol (Invitrogen) according to the manufacturer's instructions, and the quality of the isolated RNA was assessed by visualizing the ribosomal RNA bands after electrophoresis on a 1.0% agarose gel. The PrimeScript™ 1st Strand cDNA Synthesis Kit (TAKARA, Dalian, China) was used according to the manufacturer's instructions with 1 *μ*g of total RNA as a template to produce cDNA. Then the polymerase chain reaction (PCR) amplification was conducted using LA Taq (TAKARA) with the following conditions: 94°C for 5 min, 35 cycles of 94°C for 30 s, 50°C for 30 s, and 72°C for 1 min 30s, followed by one cycle of 72°C for 10 min. Rapid amplification of cDNA ends (RACE) was used to obtain the 5' and 3' ends of du* SERPINA1* using the SMART RACE cDNA amplification protocol (Clontech, Mountain View, CA, USA) and the 3'-Full RACE Kit (TaKaRa, Dalian, China), respectively. Gene-specific primers used for the amplification of RACE cDNA fragments were designed based on the obtained* SERPINA1* nucleotide sequence. The sequences of du* SERPINA1* was submitted to GenBank under the accession number KY471047. All the primer sequences mentioned above are shown in Table 1.

### 2.4. Bioinformatics Analysis

Bioinformatic analysis of du SERPINA1 was performed using a software program like DNASTAR, the NCBI website (http://www.ncbi.nlm.nih.gov) and BLAST analysis (https://blast.ncbi.nlm.nih.gov/Blast.cgi). Multiple sequence alignment was created by AlignIR V2.0 and CLC Sequence Viewer 6 was performed to construct multiple sequence alignments of the amino acid sequences of du SERPINA1 proteins. The neighbor-joining phylogenetic tree was constructed based on the alignment result using the Neighbour-Joining (NJ) algorithm within the MEGA 6.0 program.

### 2.5. Quantitative Real-Time Polymerase Chain Reaction

Total RNA was extracted from different tissues using TRIzol (Invitrogen), and 1 *μ*g total RNA was used with FastQuant RT Kit (With gDNase) (Tiangen, Beijing, China) during reverse transcription. The process included an initial phase at 42°C for 3 min, then incubation at 42°C 15 min, and incubation at 95°C for 3 min. The cDNA was stored at -80°C. Real-time qPCR was carried out on Applied Biosystems 7500 Real-Time PCR System (ThermoFisher Scientific, Waltham, MA, USA) with the following program: 1 cycle at 95°C for 5 min, followed by 40 cycles of 94°C for 30 sec, 60°C for 30 sec, and 72°C for 30 sec, and a final incubation at 72°C for 10 min. Relative quantitative of gene expression was calculated using the 2^−ΔΔCt^ method [19]. The GAPDH gene was used as an internal standard for relative expression levels. In addition, at each time point, the mean ΔCt value of control ducks was used to calibrate analysis of expression patterns in infected ducks. Therefore the results calibrated by the expressions of control ducks are displayed in the plot of du* SERPINA1 *expression of liver and kidney after DHV-1 and poly I: C challenge. All the primers are listed in Table 1.

### 2.6. Enzyme-Linked Immunosorbent Assay (ELISA) Analysis

For ELISA analysis, by using double antibody sandwich method to determine the levels of IFN-*α*, IFN-*γ*, IgG and IL-8, duck blood were gathered, and supernatants were collected after centrifugation at 3000 rpm for 20 min at 4°C and subjected to ELISA for detection of duck IFN-*α*, IFN-*γ*, IgG and IL8. The concentrations of IFN-*α*, IFN-*γ*, IgG and IL8 in the samples were measured with a multifunctional microplate reader (Tecan Infinite M200 PRO; Switzerland) and determined by comparing the optical density of the samples to the standard curve.

### 2.7. Statistical Analysis

Data were analyzed using SPSS 22.0, and differences among tissue samples were assessed with one-way ANOVA. P values less than 0.05 were considered as significant. All data were processed by GraphPad Prism 5.0.

## 3. Results

### 3.1. du SERPINA1 cDNA Sequence and Bioinformatics Analysis of SERPINA1 in Ducks

The full-length cDNA sequence of du* SERPINA1 *was 1457 bp in size and contained 47-bp 5' UTR, 135-bp 3' UTR. The open reading frame was 1275 bp and encoded a single open reading frame of 424 amino acid (aa) residues without a stop codon. The sequence has been submitted to GenBank (GenBank accession numbers: KY471047).

**Figure 1:**
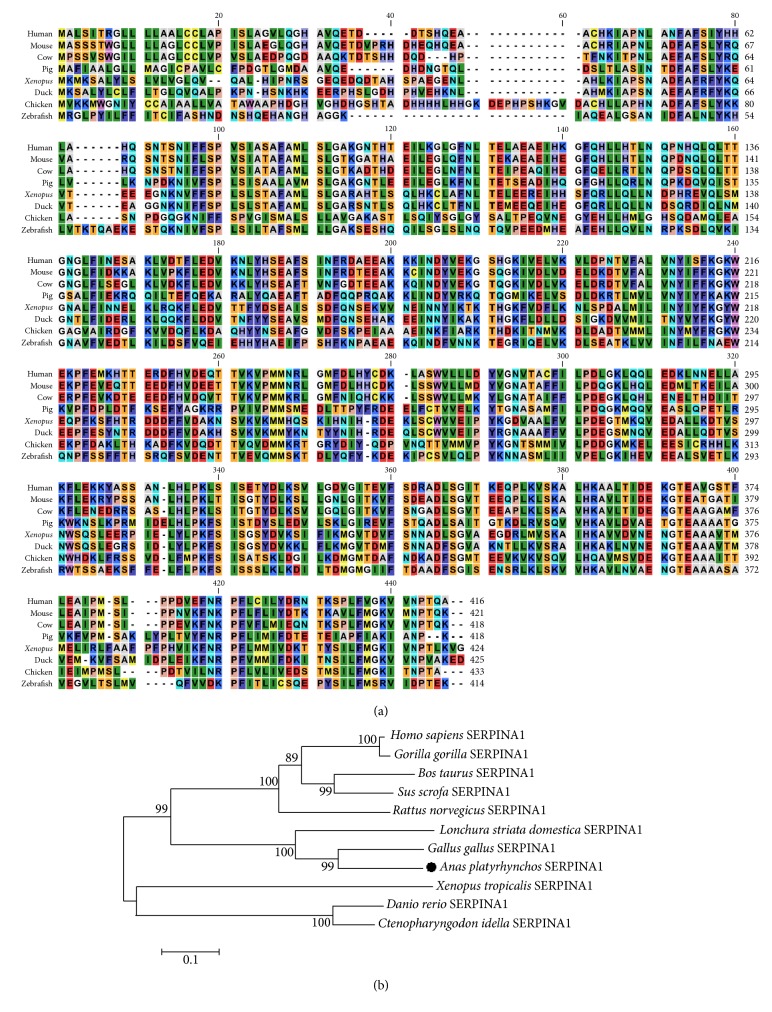
(a) Multiple alignments of SERPINA1 protein sequences from human, mouse, cow, pig, xenopus, duck, chicken and zebrafish. (b) Phylogenetic analysis of avian, mammalian, and fish SERPINA1 was carried out. The tree was constructed by the neighbor-joining tree method using amino acid sequences aligned with MEGA6. The bar indicates the bootstrap value (%). The species names and GenBank accession numbers of the SERPINA1 sequences shown are as follows: Homo sapiens, NM_001127707.1; Gorilla gorilla gorilla, XM_019009748.1; Bos taurus, NM_173882.2; Sus scrofa, NM_001348942.1; Rattus norvegicus, NM_022519.2; Lonchura striata domestic, NM_001127706.1; Gallus gallus, NM_001277493.1; Anas platyrhynchos, KY471047; Xenopus tropicalis, NM_001011275.1; Danio rerio, NM_001077758.1; Ctenopharyngodon idella, EU621405.1.

Comparing of the full-length aa sequence of du SERPINA1 to the SERPINA1 gene of other species by CLC Sequence Viewer 6, multiple alignments and amino acid sequence are shown in Figure 1(a). The duck SERPINA1 shared high similarity with SERPINA1 proteins from other vertebrates. Compared to chickens protein from the GenBank database, the du SERPINA1 protein had identities of 99 %. A condensed phylogenetic tree was constructed based on the derived amino acid sequences of SERPINA1 of organisms (Figure 1(b)). The overall tree topology revealed the following three major groups: bird, mammal and fish.

### 3.2. Tissue-Specific Expression Profile of du SERPINA1 Gene

**Figure 2:**
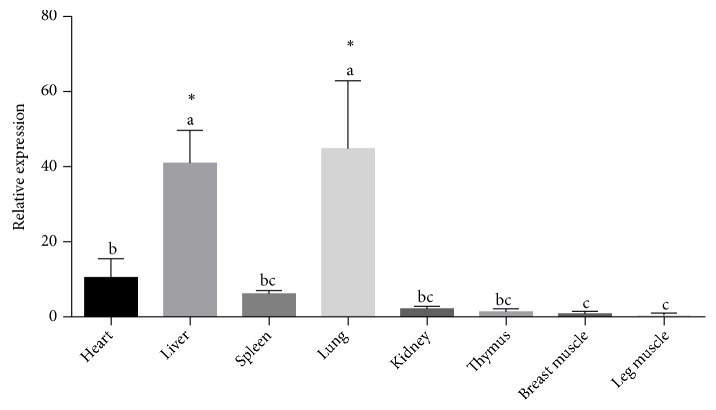
du* SERPINA1* mRNA expression in various tissues (liver, spleen, lung, heart, kidney, thymus, breast muscle and leg muscle). All assays were repeated at least three times, and data are shown as mean ± S.E. (n = 5) from one representative experiment. The expression of* SERPINA1 *was normalized to* GAPDH*. Different letters showed significant difference (*p* < 0.05).

Real-time qPCR was carried out to determine the tissue-specific expression profile of du SERPINA1 gene in healthy ducks. The results showed that du* SERPINA1 *mRNA was widely expressed in all 8 tissues tested (liver, spleen, lung, heart, kidney, thymus, breast muscle and leg muscle). The expression levels of du* SERPINA1 *mRNA were more significant higher in lung and liver tissues, whereas the expression level in other tissues was extremely low (Figure 2).

### 3.3. The Expression of Cytokine following DHV-1 and Poly I:C Treatment

**Figure 3:**
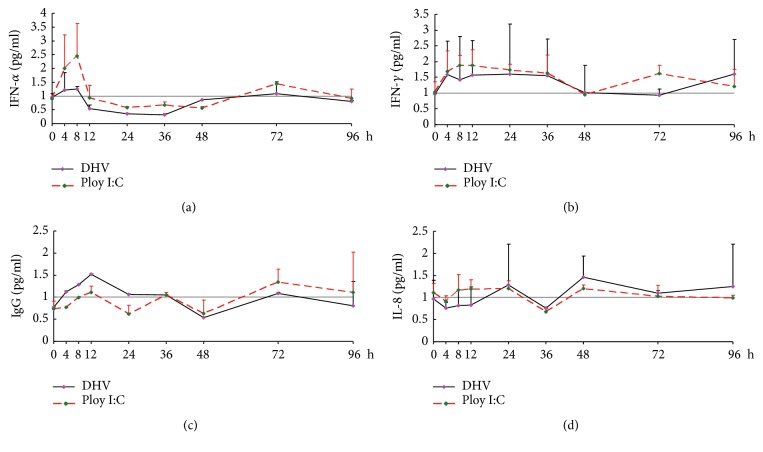
*Effect of DHV-1 and poly I:C treatment on the cytokine in the duckings*. All assays were repeated at least three times, and data are shown as mean ± S.E. (n = 5) from one representative experiment. The expression is shown for (a) IFN-*α*, (b) IFN-*γ*, (c) IgG and (d) IL-8.

In order to investigate the changes of cellular immunity and humoral immunity in ducklings during DHV-1 and poly I:C infection, the ELISA analysis was used to detect the changes of serum IgG, IFN-*α* and other cytokines. The results showed that the cytokines, including IFN-*α* and IFN-*γ* generally indicated the fluctuating trend, which performed the trend of first increasing, then decreased, and then increased (Figures 3(a) and 3(b)), while IL8 and IgG did not change much as a whole (Figures 3(c) and 3(d)).

### 3.4. du SERPINA1 Expression after DHV-1 and Poly I:C Challenge

**Figure 4:**
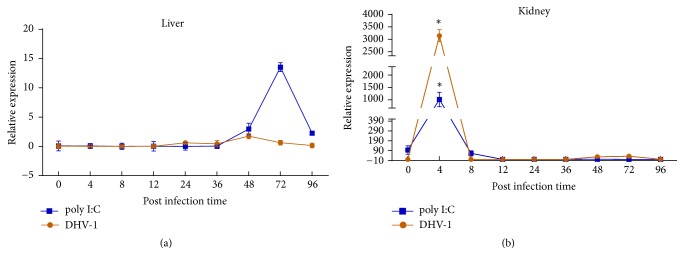
Expression of du* SERPINA1* in liver and kidney after DHV-1 or poly I:C challenge. Different letters and asterisks showed a significant difference (*p* < 0.05). The expression is shown for (a) liver and (b) kidney.

To further define the expression change of* SERPINA1 *during DHV-1 or poly I: C treatment, time-dependent expression levels in the liver and kidney after DHV-1 or poly I: C challenge were characterized (Figure 4). In the liver upon DHV-1 treatment, expression of du* SERPINA1* increased at 12 h post-infection (h.p.i), and reached peak at 48 h.p.i, after this peak, expression of du* SERPINA1* then progressively dropped. In the poly I: C treatment group, expression of du* SERPINA1* increased at 36 h.p.i, reaching a peak at 72 h.p.i, and then decreased dramatically (Figure 4(a)). The expression of du* SERPINA1* in kidney was increased immediately at 4 h.p.i with DHV-1 and poly I: C (Figure 4(b)).

## 4. Discussion

*SERPINA1*, also known as Alpha-1 anti-trypsin (AAT), is a multifunctional protein with proteinase inhibitory, anti-inflammatory and cytoprotective properties. Some studies have shown AAT could prevent the lethality of TNF or endotoxin in mice [20]. It is also illustrated in human that AAT can decrease the release of TNF-*α* from LPS stimulated and unstimulated monocytes* in vitro*. Besides, in most circulation, AAT was synthesized in the liver, and when suffering an acute phase of inflammation or infection, the AAT was released rapidly [21].

In healthy ducks, the results of tissue-specific transcriptional profile of du* SERPINA1* showed the uneven expression in different tissues. The* SERPINA1* mRNA expression in liver was significantly higher than in other tissues, supporting the fact that* SERPINA1* was attributed to different roles in physiology, particularly including immune responses associated with liver disease, which was similar with the expression pattern of SERPINA1 gene in human and mice [22, 23]. Besides, the high expression of* SERPINA1 *was also existed in lung, which was consistent with previous studies that SERPINA1 may attenuate inflammation in ventilator-induced lung injury by modulating inflammation-related protein expression [24, 25].

Cytokines can regulate the physiological functions of leukocytes, mediate inflammatory responses, participate in immune responses, and repair tissue. In order to further study the changes of host immune and inflammatory response caused by DHV-1 and poly I: C infection, the levels of cytokines in serum were tested during the process of infection. IFN-*α*, IFN-*γ*, as immunomodulatory pleiotropic factors, have been used as an indicator of cell-mediated immunity in infected organisms [26], and IL8 acts as an inflammatory cytokine and participates in the host's inflammatory response. In this study, IFN-*α* and IFN-*γ* got peaked at the early stage after DHV-1 and poly I:C infection, and the changes of IL8 and IgG were not significant after infection with DHV-1 and poly I:C [27, 28]. These results revealed the cells of the immune system was directly damage after viral infection and T-cell immunity played a major role in the process of DHV-1 and poly I:C infection, supplementing by B-cell immunity.

Subsequently, in order to explore the dynamic expression pattern of du SERPINA1 in ducks during viral infection, the du SERPINA1 mRNA expression after DHV-1 or poly I: C challenge was detected. Previous studies have showed that DHV-1 rather than other DHV types could specifically infect duck embryo liver and duck embryo kidney cells [29, 30] and in this study, liver and kidney tissues were selected to be candidate tissues. During the early period of viral injection, the expression of SERPINA1 was no significant difference in the liver, while it was rapidly increased in the kidney tissue, which may be related that during suffering the infection of the viral antigen, the host's various non-specific defenses, such as natural killer lymphocyte cell immunity, secrets more IFN-*α* and IFN-*γ* [31] to regulate the expression of SERPINA1 gene regulation pathway, which lead to an increase in early SERPINA1 gene expression. After achieving the peak, the expression of du* SERPINA1 *decreased to a normal level, which may be prevented excessive inflammatory reaction. In pig [32], when the expression of du* SERPINA1 *was up-regulated after PCV2 infection, the immune factor was transported to the immune related tissues through the circulation of blood, which prevented excessive inflammatory reaction and maintained the integrity and normal function of the immune tissue. With the increase of age, the immune system of ducklings is becoming more and more adaptive. Studies have shown that the immune level of 7-day-old chicken is comparable to that of adult chickens [6], and the mature immune system will produce a large number of cytokines to resist the attack of virus. These results suggested* SERPINA1* might be associated with the host defence response against viral infection. The specific mechanism is unclear but may autocrine regulation of* SERPINA1* mRNA synthesis when virus invasion.

Previous studies have proved that* SERPINA1* has a vital role in protect host tissue against injuring at sites of inflammation. Now, increasing evidence showed that* SERPINA1* (AAT) may exhibit biological activity independent of its protease inhibitor function [33, 34]. From our results, we speculated that when being attacked by virus, the host needs more* SERPINA1* which is consistent with the previous research results. To conclude, our results provide a better understanding of du SERPINA1 function in immunity during viral infection.

## 5. Conclusions

In this study, we first cloned and characterized the gene in duck and demonstrated that the du SERPINA1 gene shared high similarity with SERPINA1 proteins from other vertebrates. Transcriptional analyses showed ubiquitous expression of SERPINA1 gene in eight examined tissues. Expression analyses showed that SERPINA1 gene was significantly upregulated in vivo after DHV-1 or poly I: C stimulation. Taken together, our results provide a better understanding the dynamic expression change of SERPINA1 gene against virus and thereby provide a theoretical basis for future immune pathological studies.

## Figures and Tables

**Table 1:** Primers used in the study.

Primer name	Primer sequence (5'→3')	Annealing temperature (°C)	Application
du SERPINA1-F	ATGAAAATGAAGTCTGCACTG	55	RT-PCR
du SERPINA1-R	CTAGCCTACCTTGAGAGTTGGG		
du SERPINA1 5' Router	GTTCTTGTTACCTTCCTCCT	60	5' RACE-PCR
du SERPINA1 5' Inner	GGAGAGGCTCAAAGGA		
du SERPINA1 3' Router	AGGTGACCGCCTTATGGTTTCC	60	3' RACE-PCR
du SERPINA1 3' Inner	AATAATGCTGACCTCTCTGGAGTGGC		
qdu SERPINA1-F	AATACTAGCCAGCAAAACGAA	60	RT-qPCR
qdu SERPINA1-R	TGAGCCAAGTTTTCACCTTC		
GAPDH-F	TGCTAAGCGTGTCATCATCT	60	RT-qPCR
GAPDH-R	AGTGGTCATAAGACCCTCCA		

## Data Availability

The data used to support the findings of this study are available from the corresponding author upon request.

## Abbreviations

RT-PCR: Reverse transcription polymerase chain reaction
RACE: Rapid amplification of cDNA ends
ORF: Open reading frame
Real-time qPCR: Real-time quantitative polymerase chain reaction
DHV-1: Duck hepatitis virus
AAT: Alpha-1 anti-trypsin
ESTs: Expressed sequence tags
SSH: Suppression subtractive hybridization
NJ: Neighbour-Joining
Poly I:C: Polyinosinic acid:polycytidylic
ELISA: Enzyme-linked immunosorbent assay.
